# Trends in Immunotherapy (IO) Use and Survival Among Patients With High‐Incidence Stage IV Cancers Across the United States

**DOI:** 10.1002/jso.28084

**Published:** 2025-01-13

**Authors:** Samantha C. Warwar, Lauren M. Janczewski, Gladys M. Rodriguez, Jeffrey D. Wayne, David J. Bentrem

**Affiliations:** ^1^ Department of Surgery Northwestern University Feinberg School of Medicine Chicago Illinois USA; ^2^ Northwestern Quality Improvement, Research, & Education in Surgery (NQUIRES) Northwestern University Feinberg School of Medicine Chicago Illinois USA; ^3^ Division of Hematology and Oncology, Department of Medicine Northwestern University Feinberg School of Medicine Chicago Illinois USA; ^4^ Robert H. Lurie Comprehensive Cancer Center Northwestern University Chicago Illinois USA

**Keywords:** advanced cancer therapy, immunotherapy, melanoma, neoplasms by site, survival disparities

## Abstract

**Background and Objectives:**

IO has transformed cancer management, but its adoption in advanced cancer patients varies by tumor type. With more Stage IV patients undergoing surgery, understanding site‐specific outcomes in these challenging patients is essential. We aimed to evaluate IO use and survival trends for Stage IV cancer patients across high‐incidence cancers in the US.

**Methods:**

Patients diagnosed with Stage IV prostate, breast, melanoma, colorectal, renal, bladder, lung, or pancreas cancer were identified from the National Cancer Database (2004–2020). Cochrane–Armitage test and Kaplan–Meier methods assessed IO and overall survival across three periods: 2004–2010, 2011–2015, and 2016–2020.

**Results:**

Among 1 425 731 Stage IV cancer patients, most had lung (50.0%), pancreas (12.5%), and breast cancer (9.3%), while the least had melanoma (2.2%). From periods 1 to 3, IO use increased from 1.0% to 24.6%, notably in melanoma (9.5% to 58.5%, *p* < 0.001). Melanoma exhibited the greatest survival gains (median survival: 7.1 to 14.9 months). Absolute increases in 3‐year overall survival rates ranged from 3.4% in pancreas (1.7% to 5.1%) to 21.4% in melanoma (15.7% to 37.1%).

**Conclusions:**

Utilization of IO is tumor‐site specific and associated with improved survival rates for Stage IV cancer, with varied success across types. Variations in receipt highlight ongoing challenges to ensure equitable adoption.

## Summary

- We examined trends in IO use and survival among patients with Stage IV cancers (2004 to 2020).
- IO use and survival rates increased significantly, particularly in melanoma.
- However, disparities exist in IO adoption across cancer types and demographic groups.

**Abbreviations**
CI: confidence interval
CoC: American College of Surgeons Commission on Cancer
FDA: Food and Drug Administration
IO: immunotherapy
NCDB: National Cancer Database
OR: odds ratio

## 1 Introduction

IO marks a pivotal shift in the landscape of cancer treatment, offering the potential for improved outcomes where traditional therapies have had limited efficacy. The advancement in IO has notably enhanced the prognostic landscape for certain difficult‐to‐treat cancers, particularly significant for individuals with advanced‐stage cancer who frequently face limited effective treatment options and poor prognosis [1]. Important milestones for metastatic melanoma began with the Food and Drug Administration (FDA) approval of ipilimumab (Yervoy), a CTLA‐4 inhibitor, in 2011 [2], later augmented by the addition of PD‐1 inhibitors in 2014 [3]. These breakthroughs have led to a substantial increase in the 3‐year survival rate for patients with advanced melanoma, escalating from 22% in 2010–2012 to 34% in 2015–2017 [4]. Similarly, data shows survival gains in advanced lung cancer associated with recent immunotherapeutic advances [5, 6].

Surgeons are often involved in the complex management of surgical emergencies in the advanced cancer patient population. An individualized approach to surgical management is recommended, requiring surgeons to consider factors such as the patient's cancer prognosis, available treatment options, and frailty [7]. Understanding the evolving survival trends and IO use for Stage IV cancers can inform surgical decisions, which is essential for surgeons to deliver personalized care effectively. For instance, improved survival outcomes associated with IO might make aggressive surgical interventions more viable, as patients may have a better prognosis and greater potential for long‐term recovery. However, current real‐world data on the integration of IO and the trends in survival of high‐incidence advanced cancers are not sufficiently characterized, highlighting a critical gap in knowledge that this study aims to address.

In this study, we use the National Cancer Database to examine IO use and survival trends among patients diagnosed with Stage IV cancers of the prostate, breast, melanoma, colorectal, renal, bladder, lung, and pancreas from 2004 to 2020. By examining cancer‐type cohorts across three distinct time periods (year of cancer diagnosis: 2004–2010, 2011–2015, and 2016–2020), we aim to elucidate the trends in the adoption of IO and overall survival across these cancer types over time.

## 2 Materials and Methods

### 2.1 Data Source and Study Population

The National Cancer Database (NCDB) is a national cancer data registry sponsored by the American College of Surgeons Commission on Cancer (CoC) and the American Cancer Society [8]. The NCDB has been collecting clinical information on patients with malignancies since 1988, and since then, all CoC‐accredited hospitals have been required to submit data on all cancer cases [9]. Currently, data are collected from over 1500 hospitals, accounting for 72% of annual cancer cases in the United States [10]. Data are evaluated electronically for completeness and regularly audited for quality assurance and reliability [11].

From 2004 to 2020, a total of 12 422 041 patients across the specified cancer types (prostate, breast, melanoma, colorectal, renal, bladder, lung, and pancreas) were recorded in the NCDB. Among these, 1 682 538 patients were diagnosed with Stage IV cancer at the onset (excluding 10 739 503 patients diagnosed with cancer stage other than Stage IV). Stage was determined based on American Joint Committee on Cancer definitions used in the year the case was diagnosed. Patients were excluded from the study if their diagnostic data (*n* = 66 935), survival data (*n* = 106 141), or systemic treatment data (*n* = 50 764) were missing. Additionally, for colorectal and pancreatic cancers, patients with histologic subtypes other than adenocarcinoma, as defined by ICD‐O‐3 morphology codes, were excluded (colorectal *n* = 8046; pancreas *n* = 24 921). The resulting analytic cohort included 1 425 731 patients with Stage IV cancer.

Patients were categorized into primary tumor site cohort groups, with sub‐cohorts organized into three periods based on the year of advanced cancer diagnosis: 2004–2010, 2011–2015, and 2016–2020. For the survival analysis, patients diagnosed after 2019 were excluded to ensure a minimum follow‐up duration of 3 years.

### 2.2 Primary Outcome and Other Covariates

The primary outcomes were overall survival and the utilization of IO. Survival events were measured from the date of diagnosis to the date of death or last contact. To compare IO for advanced cancers over time, we calculated the number of patients with a specific cancer type who received IO and divided it by the total number of patients diagnosed with that cancer type during the same period. Additionally, we evaluated receipt of chemotherapy, hormone therapy, and surgical resection (all dichotomized into received vs not received as first‐course therapy). Biopsies that entirely removed the tumor or left only microscopic margins were classified as “surgical resection.”

Other variables included patient age, sex, race (White, Black, Hispanic, Asian, Other), income, insurance status (Private, Medicare, Medicaid, Other Government, Uninsured), and facility type. Income was reported as median ZIP code estimates based on census data and subsequently divided into quartiles. Facility type was categorized based on CoC definitions of Academic/Research Program, Integrated Network Cancer Program, Comprehensive Community Cancer Program, and Community Cancer Program. Hormone receptor assays were collected for patients with breast cancer and categorized as ER + , PR + , HER2 + , and ER‐/PR‐/HER2‐.

### 2.3 Statistical Analysis

Time‐trend analysis of IO use from 2004 to 2020 was assessed by Cochrane–Armitage test for trend. Trends in IO use among patients with Stage IV cancers over time were additionally evaluated by plotting the percentage of patients treated with IO within each time period group during the study period. A multivariable logistic regression model was constructed to assess factors associated with IO use among Stage IV cancer patients. Variables were selected based on a priori selection based on clinical validity.

Overall survival was estimated using the Kaplan–Meier method and compared between patient groups using log‐rank test. Survival curves of the entire cohort were plotted by time period. To compare survival trends among cancer types, 3‐year survival rates for each tumor site were plotted from 2004 to 2019.

Absolute differences in IO and survival rates between periods 1 and 3 were calculated for comparison across tumor sites. Median survival months in period 3 were divided by median survival months in period 1 for each tumor site to determine and compare the magnitude of change.

Level of significance was 0.05. All statistical analyses were performed using Stata version 14.2 (Stata Corporation, College Station, TX). This study utilized deidentified patient data and was determined to be exempt from review by the Northwestern University Institutional Review Board.

## 3 Results

Of 1 425 731 patients with Stage IV cancer (mean age at diagnosis 66.9 years), 783 157 were male (54.9%), and 1 068 196 were White (74.9%). The most prevalent cancer type was lung cancer (*n* = 713 370; 50.0%), followed by pancreatic (*n* = 177 686; 12.5%), breast (*n* = 133 027; 9.3%), colorectal (*n* = 130 736; 9.2%), prostate (*n* = 123 270; 8.6%), renal (*n* = 79 256; 5.6%), bladder (*n* = 37 340; 2.6%), and least common was melanoma (*n* = 31 046; 2.2%). Most patients had Medicare insurance (*n* = 772 841; 54.2%). Many patients were treated in Community Cancer Programs (*n* = 551 919; 38.7%), followed by Academic/Research facilities (*n* = 458 382; 32.2%). Further demographic details are shown in Table 1.

**Table 1:** Demographics and characteristics of patients diagnosed with Stage IV cancer by tumor site.

Characteristics	All	Prostate	Breast	Melanoma	Colorectal	Kidney	Bladder	Lung	Pancreas
*N* (%)	1 425 731 (100.0)	123 270 (8.6)	133 027 (9.3)	31 046 (2.2)	130 736 (9.2)	79 256 (5.6)	37 340 (2.6)	713 370 (50.0)	177 686 (12.5)
Median follow up time (months)	7.8	28.9	25.3	9.1	16.0	9.4	7.2	5.4	3.8
Age at diagnosis in years, mean (SD)	66.9 (12.0)	70.2 (10.7)	62.1 (14.1)	64.7 (14.6)	63.8 (13.3)	65.1 (12.0)	70.1 (11.8)	67.7 (11.2)	67.8 (11.3)
Sex									
Male	783 157 (54.9)	123 270 (100.0)	1874 (1.4)	20 989 (67.6)	74 272 (56.8)	53 592 (67.6)	25 967 (69.5)	388 526 (54.5)	94 667 (53.3)
Female	642 574 (45.1)		131 153 (98.6)	10 057 (32.4)	56 464 (43.2)	25 664 (32.4)	11 373 (30.5)	324 844 (45.5)	83 019 (46.7)
Race/Ethnicity									
White	1 068 196 (74.9)	85 419 (69.3)	92 193 (69.3)	28 325 (91.2)	94 256 (72.1)	60 465 (76.3)	29 878 (80.0)	545 684 (76.5)	131 976 (74.3)
Black	175 869 (12.3)	21 329 (17.3)	21 897 (16.5)	479 (1.5)	17 084 (13.1)	7289 (9.2)	3454 (9.3)	82 518 (11.6)	21 819 (12.3)
Hispanic	66 176 (4.6)	7792 (6.3)	7979 (6.0)	849 (2.7)	8463 (6.5)	5860 (7.4)	1503 (4.0)	23 824 (3.3)	9906 (5.6)
Asian	40 292 (2.8)	2955 (2.4)	4042 (3.0)	168 (0.5)	4319 (3.3)	1627 (2.1)	667 (1.8)	21716 (3.0)	4798 (2.7)
Other	75 198 (5.3)	5 775 (4.7)	6 916 (5.2)	1 225 (3.9)	6614 (5.1)	4015 (5.1)	1838 (4.9)	39 628 (5.6)	9187 (5.2)
Median income									
Quartile 1 (Lowest)	236 789 (16.6)	20 215 (16.4)	22 260 (16.7)	3689 (11.9)	22 032 (16.9)	12 405 (15.7)	5754 (15.4)	123 951 (17.4)	26 483 (14.9)
Quartile 2	285 775 (20.0)	23 246 (18.9)	25 289 (19.0)	6099 (19.6)	25 945 (19.8)	16 293 (20.6)	7539 (20.2)	148 389 (20.8)	32 975 (18.6)
Quartile 3	321 211 (22.5)	26 719 (21.7)	29 392 (22.1)	7097 (22.9)	28 756 (22.0)	18 294 (23.1)	8486 (22.7)	163 054 (22.9)	39 413 (22.2)
Quartile 4 (Highest)	420 414 (29.5)	37 544 (30.5)	41 144 (30.9)	10 347 (33.3)	38 967 (29.8)	22 919 (28.9)	11 232 (30.1)	200 528 (28.1)	57 733 (32.5)
Unknown	161 542 (11.3)	15 546 (12.6)	14 942 (11.2)	3814 (12.3)	15 036 (11.5)	9345 (11.8)	4329 (11.6)	77 448 (10.9)	21 082 (11.9)
Insurance									
Private	431 889 (30.3)	32 606 (26.5)	51 595 (38.8)	10 925 (35.2)	49 277 (37.7)	28 553 (36.0)	8715 (23.3)	195 003 (27.3)	55 215 (31.1)
Medicare	772 841 (54.2)	72 546 (58.9)	54 778 (41.2)	15 240 (49.1)	57 910 (44.3)	38 275 (48.3)	23 433 (62.8)	410 111 (57.5)	100 548 (56.6)
Medicaid	116 033 (8.1)	8545 (6.9)	16 018 (12.0)	2387 (7.7)	12 723 (9.7)	6016 (7.6)	2652 (7.1)	56 693 (8.0)	10 999 (6.2)
Other Government	19 954 (1.4)	1947 (1.6)	1151 (0.9)	551 (1.8)	1576 (1.2)	1137 (1.4)	539 (1.4)	10 923 (1.5)	2130 (1.2)
Uninsured	57 081 (4.0)	4991 (4.1)	6579 (5.0)	1410 (4.5)	6783 (5.2)	3226 (4.1)	1308 (3.5)	27 389 (3.8)	5395 (3.0)
Unknown	27 933 (2.0)	2635 (2.1)	2906 (2.2)	533 (1.7)	2467 (1.9)	2049 (2.6)	693 (1.9)	13 251 (1.9)	3399 (1.9)
Year of diagnosis									
2004–2010	447 381 (31.4)	29 648 (24.1)	38 512 (29.0)	8035 (25.9)	38 058 (29.1)	24 073 (30.4)	11 011 (29.5)	246 790 (34.6)	51 254 (28.8)
2011–2015	455 999 (32.0)	34 467 (28.0)	43 184 (32.5)	9975 (32.1)	42 321 (32.4)	25 387 (32.0)	12 468 (33.4)	233 113 (32.7)	55 084 (31.0)
2016–2020	522 351 (36.6)	59 155 (48.0)	51 331 (38.6)	13 036 (42.0)	50 357 (38.5)	29 796 (37.6)	13 861 (37.1)	233 467 (32.7)	71 348 (40.2)
Facility									
Academic/Research	458 382 (32.2)	45 412 (36.8)	40 414 (30.4)	11 171 (36.0)	42 702 (32.7)	30 324 (38.3)	13 181 (35.3)	210 511 (29.5)	64 667 (36.4)
Integrated Network Cancer Program	279 130 (19.6)	22 501 (18.3)	25 374 (19.1)	5579 (18.0)	24 906 (19.1)	14 323 (18.1)	7224 (19.3)	144 081 (20.2)	35 142 (19.8)
Comprehensive Community Cancer Program	113 963 (8.0)	10 057 (8.2)	10 146 (7.6)	1 873 (6.0)	10 901 (8.3)	5263 (6.6)	2855 (7.6)	61 301 (8.6)	11 567 (6.5)
Community Cancer Program	551 919 (38.7)	45 214 (36.7)	48 756 (36.7)	10 572 (34.1)	48 852 (37.4)	27 725 (35.0)	13 766 (36.9)	292 145 (41.0)	64 889 (36.5)
Unknown	22 337 (1.6)	86 (0.1)	8337 (6.3)	1851 (6.0)	3375 (2.6)	1621 (2.0)	314 (0.8)	5332 (0.7)	1421 (0.8)
Hormone receptor assays^a^									
ER+^b^			70 608 (69.3)						
PR+^c^			56 037 (55.0)						
HER2+^d^			15 935 (15.7)						
ER‐/PR‐/HER2‐ (triple negative)^e^			9 123 (9.0)						

Abbreviation: SD = standard deviation.

a Metric reported as *N* = number of patients with a particular characteristic (number of patients with a particular characteristic/number of patients with available data for the characteristic, %).

^b^ *N* = 31 187 observations with missing data.

^c^ *N* = 31 187 observations with missing data.

^d^ *N* = 31 662 observations with missing data.

^e^ *N* = 31 662 observations with missing data.

### 3.1 Trends in Immunotherapy

**Figure 1:**
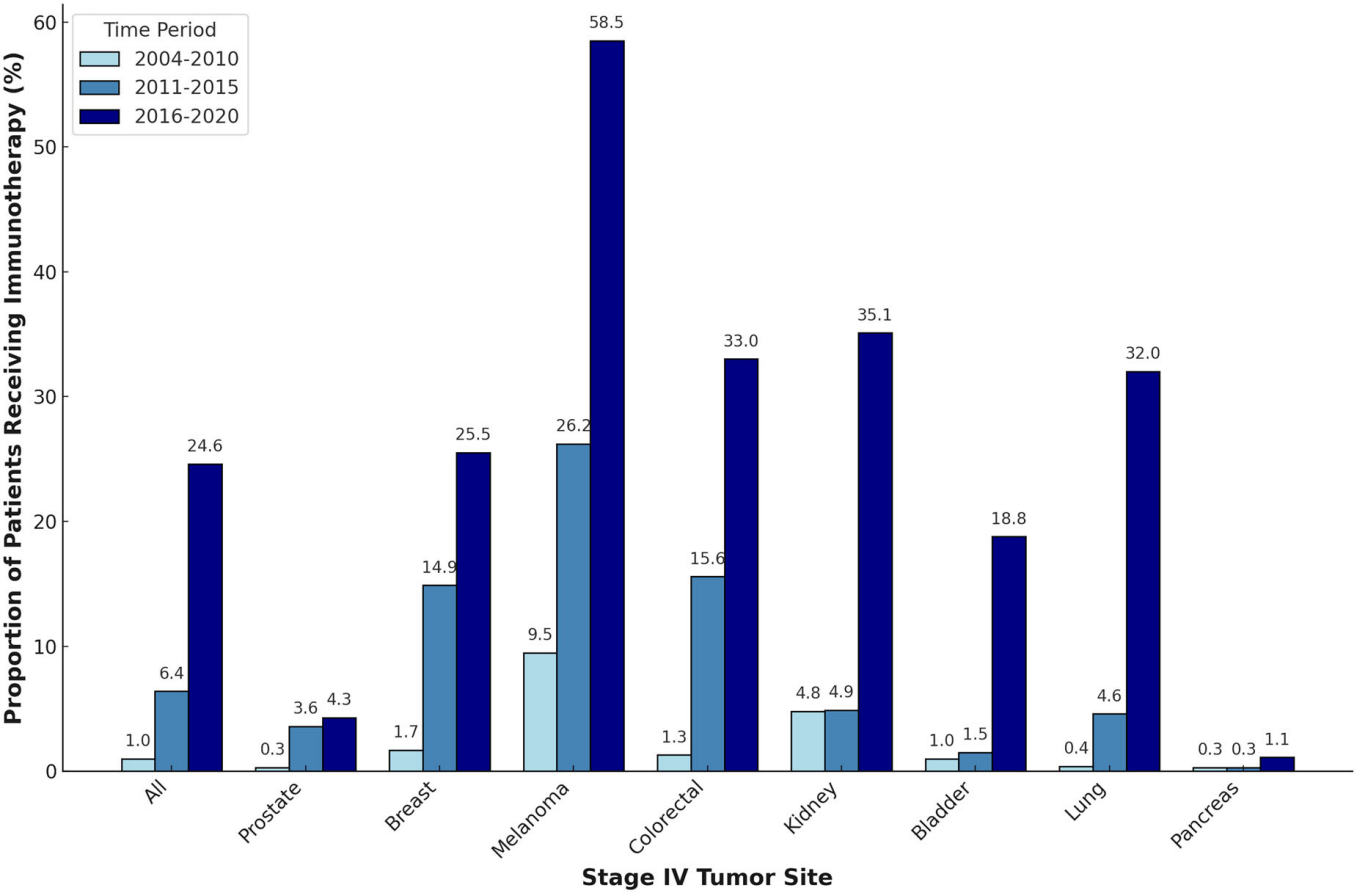
Proportion of patients receiving immunotherapy by Stage IV tumor site.

A total of 11.4% of patients with Stage IV cancer received IO between 2004 and 2020, with a notable increase over time. In period 1, only 1.0% of patients received IO. This figure rose to 6.4% in period 2 and increased to 24.6% in period 3 (*p* < 0.001, Figure 1). The most significant increase was in melanoma, where IO use surged from 9.5% in period 1% to 58.5% in period 3 (*p* < 0.001). Notable, though smaller, gains in IO use were also noted in colorectal, lung, and renal cancer, with approximately a 30% rise over time in each (colorectal: 1.3% in period 1% to 33.0% in period 3, *p* < 0.001; lung: 0.4% in period 1% to 32.0% in period 3, *p* < 0.001; renal: 4.8% in period 1% to 35.1% in period 3, *p* < 0.001; Figure 1). Breast cancer had a steady rise in IO use from 1.7% in period 1% to 25.5% in period 3 (*p* < 0.001). Pancreatic cancer showed the least uptake of IO over time, increasing from 0.3% in period 1% to 1.1% in period 3 (*p* < 0.001). This was closely followed by prostate cancer, which experienced a slight rise from 0.3% in period 1% to 4.3% in period 3 (*p* < 0.001).

The timing of maximal growth in IO use varied by cancer site. The most significant surge in IO use occurred after 2010 in melanoma, colorectal, and breast cancer. Conversely, IO use surged in lung, renal, and bladder cancer after 2015 (Figure 1). For instance, the IO rate in renal cancer remained unchanged in periods 1 and 2 (4.8% to 4.9%), then experienced a steep upward trend in period 3 (35.1%). In period 1, renal cancer had the second highest IO rate, which then fell behind the IO rates in colorectal and breast cancer in period 2, only to return to the second highest IO rate in period 3.

From 2004 to 2020, 52.0% of patients with Stage IV cancer received chemotherapy, 12.8% received hormone therapy, and 13.0% underwent surgical resection (Table 2). Trends in chemotherapy use varied by cancer type. For example, melanoma had a 22.7% decrease in chemotherapy from periods 1 to 3 (37.1% to 14.4%), while prostate cancer experienced a 12.8% increase in chemotherapy (6.6% to 19.4%). The proportion of patients receiving hormone therapy increased over time for both prostate (75.8% in period 1% to 88.2% in period 3) and breast cancers (47.0% to 57.9%). There was a decrease in the proportion of patients receiving surgical resection over time, most prominent in breast cancer, where surgical rates halved from period 1 to period 3 (31.3% to 15.7%).

**Table 2:** The proportion of patients receiving first‐course therapies for Stage IV cancer by tumor site.

Treatment	All	Prostate	Breast	Melanoma	Colorectal	Kidney	Bladder	Lung	Pancreas
*N* (%)	1 425 731	123 270 (8.6)	133 027 (9.3)	31 046 (2.2)	130 736 (9.2)	79 256 (5.6)	37 340 (2.6)	713 370 (50.0)	177 686 (12.5)
Immunotherapy, %^a^									
2004–2010	1.0	0.3	1.7	9.5	1.3	4.8	1.0	0.4	0.3
2011–2015	6.4	3.6	14.9	26.2	15.6	4.9	1.5	4.6	0.3
2016–2020	24.6	4.3	25.5	58.5	33.0	35.1	18.8	32.0	1.1
Total	11.4	3.1	15.2	35.4	16.7	16.2	7.8	12.1	0.6
Absolute difference in immunotherapy rates between period 1 and 3 ([2004–2010 IO rate]–[2016‐2020 IO rate])	23.6	4.0	23.8	49.0	31.7	30.3	17.8	31.6	0.8
Chemotherapy, %^a^									
2004–2010	51.9	6.6	54.7	37.1	68.7	45.2	52.8	54.4	56.3
2011–2015	53.8	13.8	53.1	26.4	73.9	55.7	56.3	55.7	59.5
2016–2020	50.7	19.4	65.6	14.4	74.9	42.5	48.8	50.6	59.5
Total	52.0	14.7	58.4	24.1	72.8	47.6	52.5	53.6	58.6
Hormone therapy, %^a^									
2004–2010	9.7	75.8	47.0	1.1	0.4	0.6	0.5	0.9	0.3
2011–2015	12.0	82.7	55.5	1.1	0.4	0.5	0.4	0.7	0.3
2016–2020	16.0	88.2	57.9	0.6	0.3	0.3	0.4	0.6	0.2
Total	12.8	83.7	54.0	0.9	0.3	0.5	0.4	0.8	0.3
Surgical resection, %^a^									
2004–2010	13.8	15.1	31.3	25.3	46.1	39.6	76.7	2.8	1.4
2011–2015	13.8	14.5	25.1	23.2	43.0	39.8	77.8	2.5	1.4
2016–2020	11.6	12.5	15.7	20.6	34.9	31.5	74.0	1.9	1.1
Total	13.0	13.7	23.3	22.6	40.8	36.6	76.1	2.4	1.3

a Data show the percentage of patients diagnosed with cancer site in a particular time period who received the treatment out of the total number of patients diagnosed with cancer site during the same time period.

### 3.2 Predictors of Immunotherapy Receipt

Multivariable logistic regression analysis found that patients with Stage IV cancers were less likely to receive IO if they were older (age 61–75 vs. < 51 years: odds ratio [OR] 0.92, 95% confidence interval [CI] 0.90–0.94, *p* < 0.001; age > 75 vs. < 51 years: OR 0.60, 95% CI 0.60–0.62, *p* < 0.001), insured by Medicare (vs. private: OR 0.94, 95% CI 0.92–0.95, *p* < 0.001), Medicaid (vs. private: OR 0.91, 95% CI 0.90–0.93, *p* < 0.001), or uninsured (vs. private: OR 0.56 95% CI 0.54–0.57, *p* < 0.001). Patients had higher odds of receiving IO if treated at an Academic/Research facility compared to all other facility types (Table 3). Patients with melanoma were nearly four times more likely to receive IO compared to those with lung cancer (OR 3.84, 95% CI 3.75–3.94, *p* < 0.001). See Table 3 for more details on factors associated with IO receipt.

**Table 3:** Multivariable logistic regression analysis for factors associated with immunotherapy use.

	Immunotherapy vs. no	
Predictors	OR (95% CI)	*p*‐value
Age		
< 51	Ref	
51–60	0.99 (0.97–1.01)	0.477
61–75	0.92 (0.90–0.94)	< 0.001
> 75	0.60 (0.60–0.62)	< 0.001
Sex		
Female (vs. male)	1.00 (0.98–1.01)	0.514
Insurance		
Private	Ref	
Medicare	0.94 (0.92–0.95)	< 0.001
Medicaid	0.91 (0.90–0.93)	< 0.001
Other Government	1.01 (0.97–1.06)	0.539
Uninsured	0.56 (0.54–0.57)	< 0.001
Facility		
Academic/Research	Ref	
Integrated Network Cancer Program	0.88 (0.86–0.89)	< 0.001
Comprehensive Community Cancer Program	0.86 (0.85–0.87)	< 0.001
Community Cancer Program	0.79 (0.77–0.81)	< 0.001
Cancer type		
Prostate	0.24 (0.23–0.24)	< 0.001
Breast	1.17 (1.15–1.20)	< 0.001
Melanoma	3.84 (3.75–3.94)	< 0.001
Colorectal	1.52 (1.49–1.54)	< 0.001
Kidney	1.34 (1.31–1.37)	< 0.001
Bladder	0.64 (0.61–0.66)	< 0.001
Lung	Ref	
Pancreas	0.04 (0.04–0.05)	< 0.001

Abbreviations: CI = confidence interval, OR = odds ratio.

### 3.3 Survival Trends

Table 4 shows survival analysis results. The median survival among all patients with Stage IV cancer was 8.3 months, ranging from 3.9 months in pancreatic to 38.1 months in prostate cancer. From periods 1 to 3 among all patients, median survival increased from 6.6 to 10.8 months (*p* < 0.001). An upward trend in median survival time was similarly observed in all cancer sites. Melanoma patients benefitted the most in median survival time, which more than doubled from periods 1 to 3 (7.1 to 14.9 months, *p* < 0.001). Patients with renal cancer had the second‐largest improvement in median survival, going from 8.0 months in period 1 to 13.5 months in period 3 (*p* < 0.001). Next, patients with prostate cancer, on average, lived 1.5 times longer in period 3 compared to period 1 (29.8 to 45.3 months, *p* < 0.001). The slightest improvement was seen in bladder cancer, unchanged from 7.4 months across periods.

**Table 4:** Survival among patients with Stage IV cancer by cancer type.

Survival^a^	All	Prostate	Breast	Melanoma	Colorectal	Kidney	Bladder	Lung	Pancreas
*N* (%)	1 324 934	110 516 (8.3)	122 788 (9.3)	28 404 (2.1)	120 713 (9.1)	73 340 (5.5)	34 900 (2.6)	671 387 (50.7)	162 886 (12.3)
Median survival (months)									
2004–2010	6.64	29.83	25.26	7.13	14.42	7.98	7.36	5.06	3.61
2011–2015	8.15	36.37	31.11	9.00	18.00	9.59	7.72	5.59	4.01
2016–2019	10.84	45.31	34.96	14.88	19.75	13.47	7.39	6.74	4.24
Total	8.34	38.14	30.36	9.79	17.48	10.12	7.43	5.68	3.94
Median survival magnitude of change ([2016–2019 survival months]/[2004–2010 survival months])	1.63	1.52	1.38	2.09	1.37	1.69	1.00	1.33	1.17
3‐Year survival, %									
2004–2010	13.34	44.13	38.97	15.71	21.65	16.74	12.19	6.39	1.74
2011–2015	18.88	50.36	45.32	24.43	28.11	22.44	14.84	10.50	4.14
2016–2019	26.11	56.99	49.20	37.12	31.54	29.54	16.49	17.76	5.11
Total	19.17	51.36	44.50	26.44	27.11	22.78	14.49	10.90	3.66
Absolute difference in 3‐year survival rate from period 1 and 3 ([2004–2010 3‐yr survival] – [2016–2019 3‐yr survival])	12.77	12.86	10.23	21.41	9.89	12.80	4.30	11.37	3.37
5‐Year survival, %									
2004–2010	7.88	30.08	23.83	12.12	11.25	10.26	8.34	3.24	0.94
2011–2015	12.32	35.09	30.31	19.97	16.27	15.06	11.29	6.49	2.52
2016–2019	17.74	41.15	34.18	31.14	19.17	20.31	12.57	11.42	2.98
Total	12.66	36.37	29.41	22.18	15.52	15.41	10.71	6.75	2.23
Absolute difference in 5‐year survival rate from period 1 and 3 ([2004–2010 5‐yr survival]–[2016–2019 5‐yr survival])	9.86	11.07	10.35	19.02	7.92	10.05	4.23	8.18	2.04

^a^ To ensure a minimum follow‐up time of 3 years for survival analysis, patients diagnosed after 2019 were excluded from these data.

**Figure 2:**
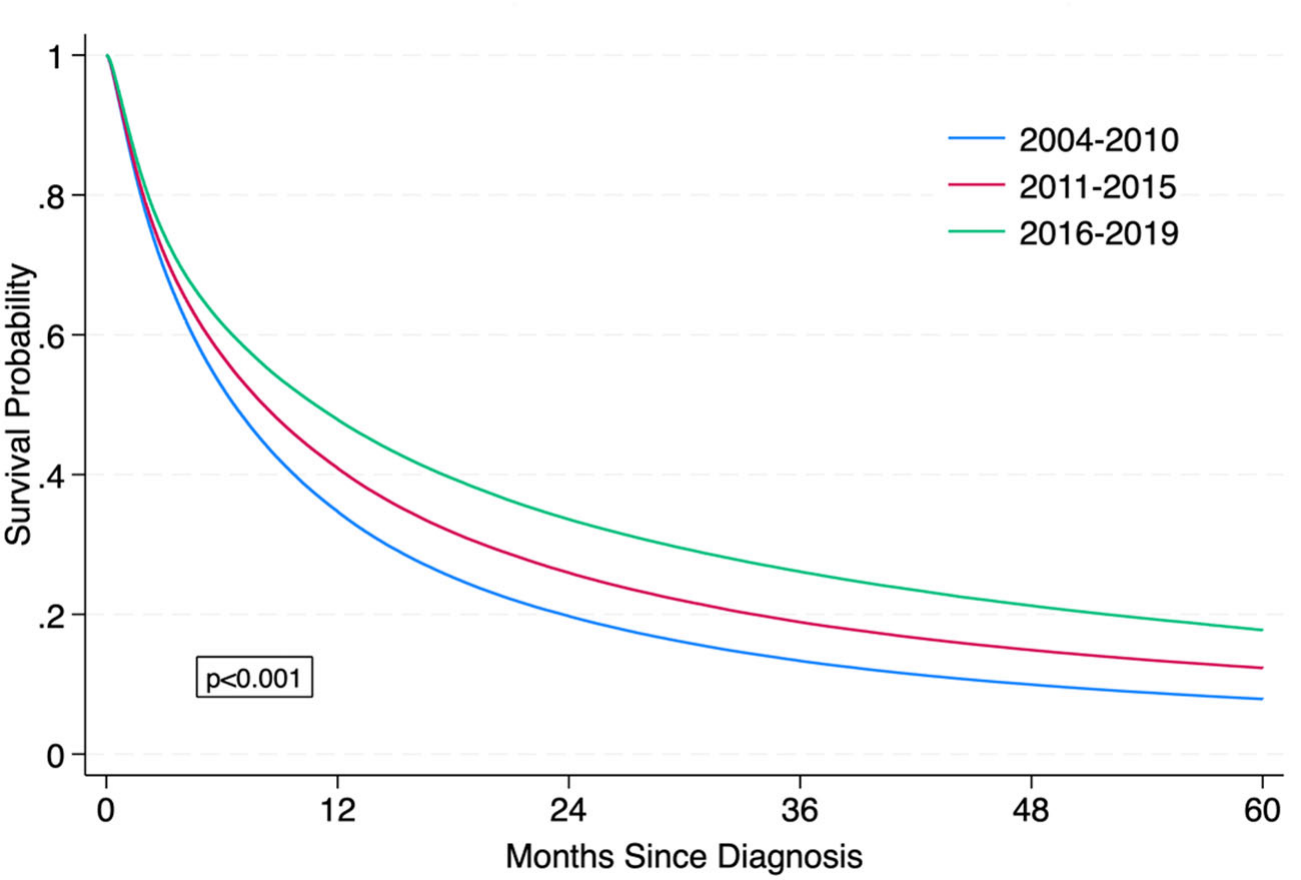
Kaplan–Meier curves estimating overall survival in patients with Stage IV cancer by time period.

**Figure 3:**
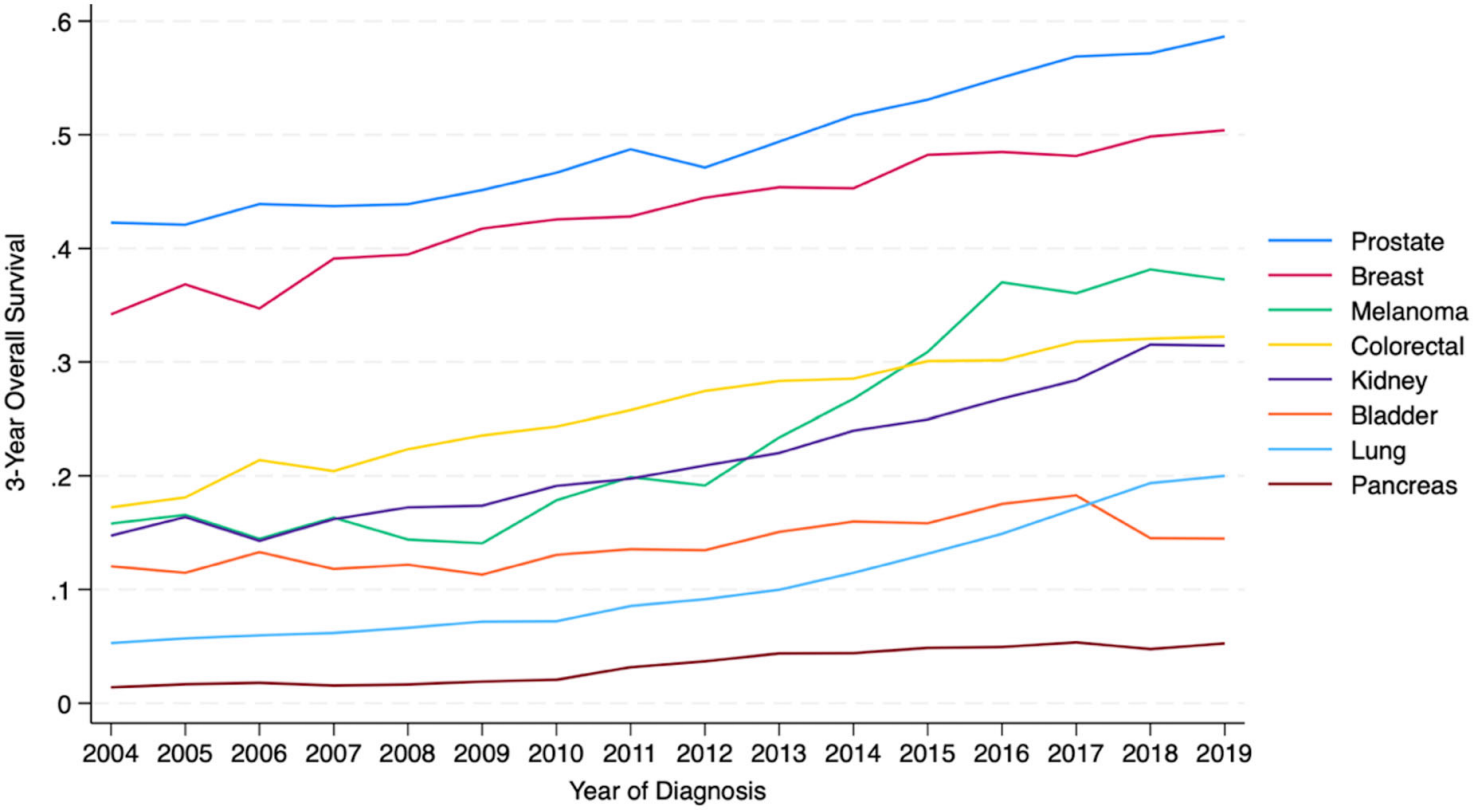
Trends in 3‐Year overall survival for patients with Stage IV cancer by tumor site.

The 3‐year survival rate among all patients with Stage IV cancer was 19.2%. Three‐year survival rates were highest in prostate (51.4%), breast (44.5%), colorectal (27.1%), melanoma (26.4%), and renal cancer (22.8%), and lowest in bladder (14.5%), lung (10.9%), and pancreas cancer (3.7%) (Table 4). Among the entire cohort, improved 3‐year survival was noted with each subsequent time frame at 13.3%, 18.9%, and 26.1% for diagnosis years 2004–2010, 2011–2015, and 2016–2019, respectively (*p* < 0.001) (Figure 2). Figure 3 depicts a comparison of 3‐year survival trends by cancer type, which shows that melanoma had the largest improvement in 3‐year survival over time. Between 2004 and 2010, melanoma's 3‐year survival rate of 15.7% was less favorable than renal at 16.7% and colorectal at 21.7%; between 2011 and 2015, melanoma's 3‐year survival rate increased to 24.4%; and between 2016 and 2019, melanoma's 3‐year survival rate rose to 37.1% surpassing renal at 29.5% and colorectal at 31.5%. Additionally, considerable improvements in 3‐year survival (reported as the absolute difference in 3‐year survival rates between periods 1 and 3) were observed in prostate cancer at 12.9%, renal at 12.8%, lung at 11.4%, and breast at 10.2% (Table 4). Pancreatic cancer had the least favorable 3‐year survival rates, with a marginal increase from 1.7% in period 1% to 5.1% in period 3.

The 5‐year survival rate among all patients with Stage IV cancer was 12.7% overall and improved from 7.9% in period 1% to 17.7% in period 3 (*p* < 0.001) (Table 4). Melanoma had a 19.0% increase in the 5‐year survival rate from periods 1 to 3 (12.1% to 31.1%, *p* < 0.001). Like 3‐year survival trends, prostate had the second largest increase in 5‐year survival from periods 1 to 3 at 11.1% (30.1% to 41.2%). Unlike 3‐year survival trends, breast cancer had the third largest improvement in 5‐year survival at 10.4% (23.8% to 34.2%), with renal cancer not far behind at 10.1% (10.3% to 20.3%). From periods 1 to 3, breast cancer was the only cancer type that improved more in 5‐year survival compared to 3‐year survival.

## 4 Discussion

Recent advances in IO have shown improved survival outcomes in clinical trials for certain advanced cancers that were previously difficult to treat. The changing prognosis among Stage IV cancer patients underscores the importance of examining trends in IO usage and their implications for survival across typical Stage IV cancers. This analysis is critical for surgeons to make well‐informed clinical decisions based on the latest outcome data. Among 1 425 731 Stage IV cancer patients, 11.4% received IO. While IO and survival rates have increased across all cancer types, some cancers have seen more substantial improvements than others.

### 4.1 Trends in Immunotherapy

From 2004 to 2020, IO usage increased from 1.0% to 24.6%. The most significant increases were observed in cases of melanoma (9.5% to 58.5%), colorectal (1.3% to 33.0%), lung (0.4% to 32.0%), and renal cancers (4.8% to 35.1%). Melanoma experienced the most significant rise, particularly after the 2011 FDA approval of ipilimumab following the MDX‐010‐20 trial, which demonstrated survival benefits with CTLA‐4 blockade [2]. Another significant increase occurred around 2014, coinciding with the FDA approval of anti‐PD1 therapies for advanced melanoma [12].

The current role of checkpoint inhibitors in the treatment of metastatic colorectal cancer is indicated for patients with microsatellite instability‐high or deficient mismatch repair tumors, which constitute only 3% to 6% of cases [13, 14]. The FDA approved Pembrolizumab (Keytruda) for these patients in 2017 [15]. Our study found that about one‐third of Stage IV colorectal cancer patients received IO, possibly influenced by the NCDB's reclassification of several monoclonal antibodies as ‘immunotherapy’ starting in 2013. As such, the NCDB IO variable includes some agents that are not checkpoint inhibitors, such as Bevacizumab (Avastin) and Cetuximab (Erbitux), which are commonly used to treat advanced colorectal cancer and thus may be contributing to the higher IO rates in our results.

Following the efficacy of immune checkpoint inhibitors in melanoma, clinical trials confirmed similar pathways for advanced lung cancer. After 2016, there was a significant increase in IO usage for Stage IV lung cancer (4.6% to 32.0%), coinciding with the FDA approval of pembrolizumab for PD‐L1 expressing tumors [16]. Before 2016, the standard treatment was platinum‐based chemotherapy [17], which decreased from 55.7% to 50.6% after 2016 in our study.

Conversely, the uptake of IO in pancreatic cancer has been minimal, highlighting the immunotherapeutic challenges due to its nonimmunogenic tumors and a suppressive tumor microenvironment dominated by immunosuppressive cells [18]. These cells create a protective niche for the tumor by inhibiting the effective immune response that could potentially destroy cancer cells. The dominance of these immunosuppressive components further reduces the efficacy of IO treatments, which rely on an active immune response.

Recognizing these immunotherapeutic challenges influences clinicians' decision‐making process regarding treatment strategies. Surgeons often care for advanced cancer patients, and they should be aware of the varying success rates of IO across diverse cancer types. This knowledge allows surgeons to effectively contribute to multidisciplinary discussions about treatment planning, especially when creating comprehensive plans involving combining surgery with chemotherapy, radiation, or other innovative therapies for cancers that do not respond well to IO.

### 4.2 Predictors of Immunotherapy Receipt

We identified socioeconomic and demographic factors associated with the underutilization of IO, aligning with existing literature. IO use was lower among older patients, those insured by Medicaid/Medicare, or uninsured, compared to younger or privately insured individuals. The literature shows that lower income levels have been linked to low IO use among patients with metastatic melanoma, highlighting financial obstacles in IO access [19, 20]. Treatment facility type also impacts IO use, with higher rates observed in academic settings [19]. A cohort study of 402 689 patients with Stage IV lung cancer, renal cell carcinoma, and melanoma demonstrated that racial and socioeconomic disparities in IO usage persisted or even widened post‐FDA approval of the first checkpoint inhibitors for treating these cancers [21]. For instance, in lung cancer, IO use was persistently lower in Black patients compared to White patients.

In contrast, in renal cell carcinoma, a new disparity in IO use among Black patients emerged following FDA approval. These disparities suggest that while IO usage is increasing, it remains unevenly accessible, particularly for those with lower socioeconomic status or in nonacademic settings. These disparities indicate that while IO usage is rising, it remains unevenly accessible, particularly for those with lower socioeconomic status or in nonacademic settings.

### 4.3 Survival Trends

Our results indicate a general improvement in survival among patients with high‐incidence Stage IV cancers. The 3‐year survival rate for Stage IV cancers improved from 13.3% in 2004–2010 to 26.1% in 2016–2019. Melanoma recorded the most significant survival enhancement, with median survival more than doubling from 7.1 months to 14.9 months, closely followed by renal cancer. These improvements align with existing literature attributing survival gains to the introduction of checkpoint inhibitors and targeted therapies [22, 23]. Both melanoma and renal cancer experienced higher IO utilization across periods compared to other cancers, supporting their notable survival outcomes.

Prostate and breast cancers maintained the best prognosis across all three analyzed periods. Notably, prostate cancer demonstrated marked improvements in both 3‐ and 5‐year survival rates despite minimal IO usage, which remained below 5% throughout the study. These survival improvements are linked to multimodal treatment advances [24, 25, 26, 27, 28, 29]. Conversely, survival rates for Stage IV pancreatic and bladder cancers remained low, consistent with historical challenges. Bladder cancer's progress has been hampered by historically contradictory study results regarding IO efficacy [30]. However, the recent Phase 3 Checkmate 901 trial, which showed that combining nivolumab (Opdivo) with chemotherapy significantly extends survival compared to chemotherapy alone, suggests a shift in treatment paradigms for metastatic bladder cancer [31]. Due to the recency of these findings, interpretations of bladder cancer survival data should be cautious, as our data set does not fully capture these latest advancements.

The variation of survival improvements among different cancer types highlights the heterogeneity. While melanoma, renal, and prostate cancers have seen substantial gains, other cancers, like pancreatic cancer, have lagged or are just now making headway, like bladder cancer. However, the minimal progress in some cancers underscores ongoing challenging areas where efforts should be made to ensure all patients benefit from these medical advancements.

The persistent disparities in survival rates among different cancer types and the modest gains in certain cancers suggest the diverse needs among the Stage IV cancer patient population.

### 4.4 Limitations

This study has some limitations. First, the NCDB does not report details regarding the specific agents, doses, schedules, duration of treatment, or associated toxicities for IO, targeted therapy, or chemotherapy. Second, as the NCDB only collects data from CoC‐accredited hospitals, there may be a selection bias as these hospitals are more likely to be larger, urban, and academically affiliated. This may not accurately reflect outcomes from smaller, rural, or nonaccredited hospitals. As a result, the findings might not apply to all Stage IV cancer patients receiving treatment in various healthcare environments. Additionally, patients diagnosed after 2019 were omitted from the survival analysis to guarantee at least 3 years of follow‐up. Therefore, any changes in clinical practice or therapy advancements that happened toward the end of the study period might not be fully represented.

## 5 Conclusion

Utilization of IO is tumor‐site specific and is associated with improved survival rates for Stage IV cancer, with varying degrees of success across cancer types. Variations in receipt highlight ongoing challenges in ensuring equitable adoption across patient and center types.

## Disclosure

The authors have nothing to disclose. All authors have approved the final article.

## Synopsis

We examined trends in IO use and survival among patients with stage IV cancers (2004 to 2020). IO use and survival rates increased significantly, particularly in melanoma. However, disparities exist in IO adoption across cancer types and demographic groups.

## Data Availability

The datasets analyzed during the current study are not publicly available, as they were provided by the American College of Surgeons. They are available to investigators in Commission on Cancer‐accredited cancer programs through an application process.

## References

[1] M. K. Callahan , M. A. Postow , and J. D. Wolchok , “Targeting T Cell Co‐Receptors for Cancer Therapy,” Immunity 44, no. 5 (2016): 1069–1078. 27192570 10.1016/j.immuni.2016.04.023

[2] F. S. Hodi , S. J. O' , D. F. McDermott , et al., “Improved Survival With Ipilimumab in Patients With Metastatic Melanoma,” New England Journal of Medicine 363, no. 8 (2010): 711–723. 20525992 10.1056/NEJMoa1003466 PMC3549297

[3] D. Schadendorf , F. S. Hodi , C. Robert , et al., “Pooled Analysis of Long‐Term Survival Data From Phase II and Phase III Trials of Ipilimumab in Unresectable or Metastatic Melanoma,” Journal of Clinical Oncology 33, no. 17 (2015): 1889–1894. 25667295 10.1200/JCO.2014.56.2736 PMC5089162

[4] K. D. Miller , L. Nogueira , T. Devasia , et al., “Cancer Treatment and Survivorship Statistics, 2022,” CA: A Cancer Journal for Clinicians 72, no. 5 (2022): 409–436. 35736631 10.3322/caac.21731

[5] T. Voruganti , P. R. Soulos , R. Mamtani , C. J. Presley , and C. P. Gross , “Association Between Age and Survival Trends in Advanced Non‐Small Cell Lung Cancer After Adoption of Immunotherapy,” JAMA Oncology 9, no. 3 (2023): 334–341. 36701150 10.1001/jamaoncol.2022.6901 PMC9880865

[6] L. Gandhi , D. Rodríguez‐Abreu , S. Gadgeel , et al., “Pembrolizumab Plus Chemotherapy in Metastatic Non‐Small‐Cell Lung Cancer,” New England Journal of Medicine 378, no. 22 (2018): 2078–2092. 29658856 10.1056/NEJMoa1801005

[7] T. E. Grotz , “Surgical Emergencies in Advanced Cancer Patients: How to Navigate a Perilous Time,” ACS Bulletin, 2021, https://www.facs.org/for‐medical‐professionals/news‐publications/news‐and‐articles/bulletin/2021/06/surgical‐emergencies‐in‐advanced‐cancer‐patients‐how‐to‐navigate‐a‐perilous‐time/.

[8] G. D. Steele , D. P. Winchester , and H. R. Menck , “The National Cancer Data Base. A Mechanism for Assessment of Patient Care,” Cancer 73, no. 2 (1994): 499–504. 8293419 10.1002/1097-0142(19940115)73:2<499::aid-cncr2820730241>3.0.co;2-t

[9] D. J. Boffa , J. E. Rosen , K. Mallin , et al., “Using the National Cancer Database for Outcomes Research,” JAMA Oncology 3, no. 12 (2017): 1722. 28241198 10.1001/jamaoncol.2016.6905

[10] K. Mallin , A. Browner , B. Palis , et al., “Incident Cases Captured in the National Cancer Database Compared With Those in U.S. Population Based Central Cancer Registries in 2012–2014,” Annals of Surgical Oncology 26, no. 6 (2019): 1604–1612. 30737668 10.1245/s10434-019-07213-1

[11] D. P. Winchester , A. K. Stewart , J. L. Phillips , and E. E. Ward , “The National Cancer Data Base: Past, Present, and Future,” Annals of Surgical Oncology 17, no. 1 (2010): 4–7. 19847564 10.1245/s10434-009-0771-3 PMC2805801

[12] L. A. Raedler , “Keytruda (Pembrolizumab): First PD‐1 Inhibitor Approved for Previously Treated Unresectable or Metastatic Melanoma,” American Health and Drug Benefits 8, no. Spec Feature (2015): 96–100. 26629272 PMC4665064

[13] K. Nosho , Y. Baba , N. Tanaka , et al., “Tumour‐Infiltrating T‐Cell Subsets, Molecular Changes in Colorectal Cancer, and Prognosis: Cohort Study and Literature Review,” Journal of Pathology 222, no. 4 (2010): 350–366. 20927778 10.1002/path.2774 PMC3033700

[14] L. H. Biller and D. Schrag , “Diagnosis and Treatment of Metastatic Colorectal Cancer: A Review,” Journal of the American Medical Association 325, no. 7 (2021): 669–685. 33591350 10.1001/jama.2021.0106

[15] G. Golshani and Y. Zhang , “Advances in Immunotherapy for Colorectal Cancer: A Review,” Therapeutic Advances in Gastroenterology 13 (2020): 175628482091752. 10.1177/1756284820917527 PMC7268115 32536977

[16] L. Pai‐Scherf , G. M. Blumenthal , H. Li , et al., “FDA Approval Summary: Pembrolizumab for Treatment of Metastatic Non‐Small Cell Lung Cancer: First‐Line Therapy and Beyond,” Oncologist 22, no. 11 (2017): 1392–1399. 28835513 10.1634/theoncologist.2017-0078 PMC5679831

[17] J. Huang and K. L. Reckamp , “Immunotherapy in Advanced Lung Cancer,” Oncology (Williston Park, NY) 34, no. 7 (2020): 272–279. 32674216

[18] R. J. Torphy , Y. Zhu , and R. D. Schulick , “Immunotherapy for Pancreatic Cancer: Barriers and Breakthroughs,” Annals of Gastroenterological Surgery 2, no. 4 (2018): 274–281. 30003190 10.1002/ags3.12176 PMC6036358

[19] N. Lamba , P. A. Ott , and J. B. Iorgulescu , “Use of First‐Line Immune Checkpoint Inhibitors and Association With Overall Survival Among Patients With Metastatic Melanoma in the Anti–PD‐1 Era,” JAMA Network Open 5, no. 8 (2022): e2225459. 36006646 10.1001/jamanetworkopen.2022.25459 PMC9412220

[20] F. Ramirez , R. Vasquez , J. Gill , et al., “Understanding the Association Between Social Determinants of Health and Receipt of Immunotherapy for Melanoma,” Journal of Drugs in Dermatology 23, no. 5 (2024): 311–315. 38709695 10.36849/JDD.7803

[21] T. Ermer , M. E. Canavan , R. C. Maduka , et al., “Association Between Food and Drug Administration Approval and Disparities in Immunotherapy Use Among Patients With Cancer in the US,” JAMA Network Open 5, no. 6 (2022): e2219535. 35771575 10.1001/jamanetworkopen.2022.19535 PMC9247736

[22] V. Shah , V. Panchal , A. Shah , B. Vyas , S. Agrawal , and S. Bharadwaj , “Immune Checkpoint Inhibitors in Metastatic Melanoma Therapy (Review),” Medicine International 4, no. 2 (2024): 13. 38410760 10.3892/mi.2024.137 PMC10895472

[23] A. Deleuze , J. Saout , F. Dugay , et al., “Immunotherapy in Renal Cell Carcinoma: The Future Is Now,” International Journal of Molecular Sciences 21, no. 7 (2020): 2532. 32260578 10.3390/ijms21072532 PMC7177761

[24] C. J. Sweeney , Y.‐H. Chen , M. Carducci , et al., “Chemohormonal Therapy in Metastatic Hormone‐Sensitive Prostate Cancer,” New England Journal of Medicine 373, no. 8 (2015): 737–746. 26244877 10.1056/NEJMoa1503747 PMC4562797

[25] J. Jiang , Y. Wang , J. Bai , et al., “Efficacy and Safety of Triple or Dual Therapies for Metastatic Hormone‐Sensitive Prostate Cancer: A Systematic Review and Bayesian Network Meta‐Analysis,” Future Oncology (2024): 1–16. 10.2217/FON-2022-1114 PMC12947570 41697112

[26] C. J. Ryan , M. R. Smith , J. S. De Bono , et al., “Abiraterone in Metastatic Prostate Cancer Without Previous Chemotherapy,” New England Journal of Medicine 368, no. 2 (2013): 138–148. 23228172 10.1056/NEJMoa1209096 PMC3683570

[27] H. I. Scher , K. Fizazi , F. Saad , et al., “Increased Survival With Enzalutamide in Prostate Cancer After Chemotherapy,” New England Journal of Medicine 367, no. 13 (2012): 1187–1197. 22894553 10.1056/NEJMoa1207506

[28] P. W. Kantoff , C. S. Higano , N. D. Shore , et al., “Sipuleucel‐T Immunotherapy for Castration‐Resistant Prostate Cancer,” New England Journal of Medicine 363, no. 5 (2010): 411–422. 20818862 10.1056/NEJMoa1001294

[29] C. Parker , S. Nilsson , D. Heinrich , et al., “Alpha Emitter Radium‐223 and Survival in Metastatic Prostate Cancer,” New England Journal of Medicine 369, no. 3 (2013): 213–223. 23863050 10.1056/NEJMoa1213755

[30] M. Larroquette , F. Lefort , C. Domblides , et al., “How Immunotherapy Has Redefined the Treatment Paradigm of Metastatic or Locally Advanced Muscle‐Invasive Urothelial Bladder Carcinoma,” Cancers 16, no. 9 (2024): 1780. 38730732 10.3390/cancers16091780 PMC11083785

[31] M. S. van der Heijden , G. Sonpavde , T. Powles , et al., “Nivolumab Plus Gemcitabine–Cisplatin in Advanced Urothelial Carcinoma,” New England Journal of Medicine 389, no. 19 (2023): 1778–1789. 37870949 10.1056/NEJMoa2309863 PMC12314471

